# Elderly patients treated with Onyx versus Orsiro drug-eluting coronary stents in a randomized clinical trial with long-term follow-up

**DOI:** 10.1007/s00392-025-02622-7

**Published:** 2025-03-04

**Authors:** Daphne van Vliet, Eline H. Ploumen, Tineke H. Pinxterhuis, Carine J. M. Doggen, Adel Aminian, Carl E. Schotborgh, Peter W. Danse, Ariel Roguin, Rutger L. Anthonio, Edouard Benit, Marlies M. Kok, Gerard C. M. Linssen, Clemens von Birgelen

**Affiliations:** 1 Department of Cardiology, Thoraxcentrum Twente, Medisch Spectrum Twente, (A-25), Koningsplein 1, 7512 KZ Enschede, The Netherlands; 2 Department of Health Technology and Services Research, Faculty of Behavioural, Management and Social Sciences, Technical Medical Centre, University of Twente, Enschede, The Netherlands; 3 Department of Cardiology, Centre Hospitalier Universitaire de Charleroi, Charleroi, Belgium; 4 Department of Cardiology, Haga Hospital, The Hague, The Netherlands; 5 Department of Cardiology, Rijnstate Hospital, Arnhem, The Netherlands; 6 Department of Cardiology, Hillel Yaffe Medical Center Hadera, and B. Rappaport-Faculty of Medicine Israel Institute of Technology, Haifa, Israel; 7 Department of Cardiology, Scheper Hospital, Treant Zorggroep, Emmen, The Netherlands; 8 Department of Cardiology, Jessa Hospital, Hasselt, Belgium; 9 Department of Cardiology, Ziekenhuisgroep Twente, Almelo, Hengelo, The Netherlands

**Keywords:** Elderly patients, Randomized controlled trial, Coronary artery disease, Percutaneous coronary intervention, Drug-eluting stents, Long-term follow-up

## Abstract

**Background:**

Percutaneous coronary intervention (PCI) with new-generation drug-eluting stents is increasingly performed in elderly patients, who generally have more comorbidities and more technically challenging target lesions. Nevertheless, there is a paucity of reported data on the *long-term* safety and efficacy of PCI with contemporary stents in elderly all-comers.

**Methods:**

This prespecified secondary analysis of a large-scale randomized clinical trial (BIONYX; *clinicaltrials.gov:NCT02508714*) compared in elderly all-comers (≥ 75 years) the 5-year outcome after PCI with the novel, more radiopaque Onyx zotarolimus-eluting stent (ZES) versus the Orsiro sirolimus-eluting stent (SES). We assessed the main composite endpoint target vessel failure (TVF: cardiac death, target vessel myocardial infarction, or target vessel revascularization) and several secondary endpoints.

**Results:**

Of 2,488 trial participants, 475(19.1%) were elderly (79.5 ± 3.5 years), including 165(34.7%) women. There was a significant between-stent difference in the main endpoint TVF in favor of the Onyx ZES (14.4% vs. 24.2%, HR: 0.60, 95% CI 0.39–0.93, p_log-rank_ = 0.02). The time-to-event curves displayed between-stent dissimilarities across all components of TVF, yet not statistically significant. Landmark analysis between 1- and 5-year follow-up showed in Onyx ZES-treated patients significantly lower rates of TVF (7.8% vs.8.9%, p = 0.002) and target vessel revascularization (3.0% vs.8.3%, p = 0.029). In addition, the 5-year rates of all-cause mortality and several composite endpoints were lower (p < 0.03) in Onyx ZES-treated patients.

**Conclusions:**

In elderly all-comer patients, those treated with Onyx ZES showed a lower 5-year incidence of the main endpoint of safety and efficacy, as well as several secondary endpoints, than patients treated with Orsiro SES. Further research on this issue is warranted.

**Clinical trial registration information:**

https://clinicaltrials.gov/study/NCT02508714.

**Graphical abstract:**

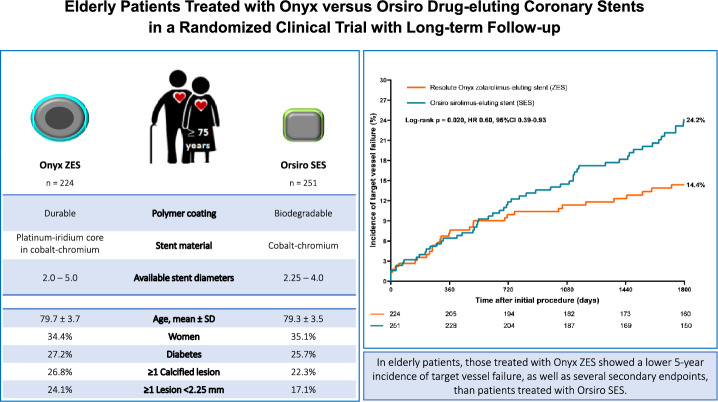

**Supplementary Information:**

The online version contains supplementary material available at 10.1007/s00392-025-02622-7.

## Introduction

Coronary artery disease is the main contributor to morbidity and mortality in aging populations with Western lifestyle [1]. In elderly patients with obstructive coronary artery disease, percutaneous coronary intervention (PCI) with contemporary drug-eluting stents (DES) is a valuable therapeutic option [2]. With advancing age, there is an increase in the prevalence of cardiovascular risk factors, associated with a higher burden and complexity of coronary atherosclerosis [3, 4]. Therefore, elderly patients more often require PCI in diffusely diseased target vessels and complex, calcified plaques. In addition, due to the presence of more comorbidities and more challenging target lesions, PCI in the elderly is associated with worse clinical outcomes [3, 5, 6].

While PCI is increasingly performed in elderly patients, there is a lack of study data about the *long-term* safety and efficacy of PCI with contemporary DES in this patient subset [7, 8]. In addition, previous studies in elderly PCI patients often focused on acute coronary syndromes and had a short follow-up [5, 9]. As life expectancy of the elderly has increased and many of them live a rather active life [10], PCI in the elderly is currently not reserved for the acute setting but increasingly used for treating chronic stable angina [11, 12]. Therefore, it is desirable that a comparison of DES types in elderly PCI patients is performed in an all-comer patient population that reflects current clinical practice.

The BIONYX trial [13] compares two contemporary DES types in all-comer PCI patients, with a substantial proportion being 75 years or older. The novel Onyx zotarolimus-eluting stent (ZES) is designed from a thin composite-wire strut that has a platinum-iridium core to enhance radiographic visibility [13–15]. These stent features may be particularly valuable in elderly all-comers who more often have a limited radiographic visibility, and challenging and calcified target lesions [16]. In the entire trial population, the Onyx stent showed no between-stent difference in clinical outcome as compared to the widely used Orsiro sirolimus-eluting stent (SES) [17, 18]. Yet, this does not exclude a potential advantage of the Onyx ZES among elderly patients [18]. Therefore, we performed a pre-specified secondary analysis of the trial in elderly patients, comparing those treated with Onyx ZES versus patients who were treated with Orsiro SES.

## Methods

### Study design and population

The present analysis was performed in all participants of the BIONYX trial (*clinicaltrials.gov: NCT02508714*), who at the time of enrolment were aged ≥ 75 years. In brief, this trial is a patient- and assessor-blinded, randomized, multicenter study, evaluating the safety and efficacy of the *Onyx* durable polymer-coated ZES versus the *Orsiro* biodegradable polymer-coated SES in an all-comer population with very few exclusion criteria. Study design, in-/exclusion criteria, and all study procedures of the trial have been described in detail [13]. Overall, 2,488 patients were enrolled in the main trial between October 2015 and December 2016. Briefly, the main trial showed similar long-term outcomes of safety and efficacy for Onyx ZES and Orsiro SES in both all-comers and diabetic patients [13, 15, 17, 18]. At 5-year follow-up, outcome data were available in 2,414 patients (follow-up rate: 97.0%). Patients were enrolled in the randomized trial after providing written informed consent. The study complied with the CONSORT 2010 Statement and Declaration of Helsinki [19, 20]. It was approved by the Medical Ethics Committee Twente (P15-19) and the institutional review boards of all participating centers.

### Randomization, procedures, and follow-up

At the time of inclusion, patients were randomly allocated to one of the two contemporary study stents: Resolute Onyx (Medtronic, Santa Rosa, CA, USA), available in diameters ranging from 2.0 to 5.0 mm, and Orsiro (Biotronik, Berlin, Germany), available in diameters ranging from 2.25 to 4.0 mm. More detailed descriptions of both study devices have previously been reported [13]. PCI was performed by experienced interventional cardiologists in consonance with current international guidelines. During 5-year follow-up, clinical events were assessed annually after the first PCI at a visit to an outpatient clinic, or by questionnaires and/or telephone follow-up. Patients and assessors were blinded to the allocated stents, while the treating physicians were not. The trial was independently monitored. Potential adverse clinical events were adjudicated by an external clinical event committee consisting of interventional cardiologists affiliated with the University of Amsterdam, the Netherlands.

### Purpose and definition of clinical outcomes

Purpose of this pre-specified secondary analysis was to assess the safety and efficacy of both study devices in elderly patients. Safety and efficacy were evaluated by the same clinical endpoints as assessed in the original clinical trial: The main endpoint target vessel failure (TVF) which is a composite of cardiac death, target vessel myocardial infarction (TVMI), or target vessel revascularization (TVR). Secondary study endpoints were: the composite endpoint target lesion failure (TLF: cardiac death, TVMI, or TVR) as well as its components; major adverse cardiac events (MACE: all-cause death, any myocardial infarction, or clinically indicated target lesion revascularization); patient-oriented composite endpoint (POCE: all-cause death, any myocardial infarction, or any revascularization) and stent thrombosis.

### Statistical analysis

To compare categorical variables, Pearson’s chi square test or Fisher’s exact test were applied, and the student t test was used for the comparison of continuous variables. Time to endpoints was assessed using Kaplan–Meier methods, and the log-rank test was applied for between-group comparison. The Cox proportional hazards regression model was used to compute hazard ratios (HR) with 2-sided confidence intervals (CI). Patients who were lost to follow-up, died, or withdrew consent were censored at the time of last contact or time of death. Two-sided P values < 0.05 were considered statistically significant. A multivariate model was constructed with the use of stepwise backward selection. Since no variables showed a significant association with TVF, the final model only consisted of the stratification factors sex and diabetes that were applied for randomization in the main trial. Landmark analyses were performed of the primary endpoint and its individual components between 1- and 5-year follow-up. Statistical analyses were performed with SPSS statistics version 24.0 (IBM Corp, Armonk, NY, USA).

## Results

### Characteristics of study population and target lesions

Of all 2,488 trial participants, 475 (19.1%) were at least 75 years old (79.5 ± 3.5 years) and assessed in the present study. Of these study patients, treated for 638 target lesions, 165 (34.7%) were women and 122 (25.7%) were known to have diabetes, of whom 39/122 (32.0%) were treated with insulin. Almost two-thirds of the study patients presented with acute coronary syndromes, while about one third was treated for stable angina.

Of all study patients, 224/475 (47.2%) had been randomly allocated to the Onyx ZES (treated for 307 target lesions), and 251/475 (52.8%) to the Orsiro SES (treated for 331 target lesions). Between the two stent groups, there was no difference in baseline medical history, clinical presentation, and procedural details, except for a higher percentage of white patients (98.2% vs. 100%, p = 0.03) treated in the Onyx group. Further baseline characteristics are presented in Table 1. In addition, at the time of hospital discharge and at 5-year follow-up, there was no significant difference between the two stent groups in the use of anticoagulant and antiplatelet therapy (Supplementary Table 1). No between-group difference was seen with regard to the available extended PCI procedural characteristics (Supplementary Table 2).

**Table 1:** Characteristics of patients, target lesions, and invasive treatment

	All patients *n* = 475	Onyx ZES *n* = 224	Orsiro SES *n* = 251	*p* value
Age, mean (year)	79.5 (3.5)	79.7 (3.7)	79.3 (3.5)	0.24
Women	165 (34.7)	77 (34.4)	88 (35.1)	0.88
White	471 (99.2)	220 (98.2)	251 (100)	*0.033*
Body-mass index, mean (kg/m^2^)	26.8 (4.1)	26.9 (4.4)	26.6 (3.7)	0.38
Medical history				
Diabetes Mellitus Insulin-dependent diabetes	122 (25.7) 39 (32.0)	61 (27.2) 23 (37.7)	61 (25.7) 16 (26.2)	0.47
Hypertension	293 (61.7)	136 (60.7)	157 (62.5)	0.66
Previous myocardial infarction	104 (21.9)	45 (20.1)	59 (23.5)	0.37
Previous stroke	74 (15.6)	37 (16.5)	37 (14.7)	0.59
Renal insufficiency	86 (18.1)	41 (18.3)	45 (17.9)	0.92
Previous PCI	118 (24.8)	60 (26.8)	58 (23.1)	0.35
Previous CABG	60 (12.6)	30 (13.4)	30 (12.0)	0.64
Current smoker	48 (10.1)	24 (10.7)	24 (9.6)	0.74
Clinical presentation				
Acute coronary syndrome				0.79
STEMI	77 (16.2)	35 (15.6)	42 (16.7)	
Non-STEMI	138 (29.1)	70 (31.3)	68 (27.1)	
Unstable Angina	94 (19.8)	42 (18.8)	52 (20.7)	
Stable Angina	166 (34.9)	77 (34.4)	89 (35.5)	
Lesion and procedural characteristics				
Treated coronary				
Left main	19 (4.0)	12 (5.4)	7 (2.8)	0.15
Left anterior descending artery	273 (57.5)	129 (57.6)	144 (57.4)	0.96
Left circumflex artery	135 (28.4)	62 (27.7)	73 (29.1)	0.74
Right coronary artery	148 (31.2)	70 (31.3)	78 (31.1)	0.97
At least 1 complex lesion	373 (78.5)	179 (79.9)	194 (77.3)	0.49
At least 1 bifurcation lesion	219 (46.1)	99 (44.2)	120 (47.8)	0.43
At least 1 chronic total occlusion	17 (3.6)	7 (3.1)	10 (4.0)	0.62
At least 1 bypass graft lesion	14 (2.9)	7 (3.1)	7 (2.8)	0.83
At least 1 severely calcified lesion	116 (24.4)	60 (26.8)	56 (22.3)	0.26
At least 1 lesion > 27 mm	106 (22.3)	49 (21.9)	57 (22.7)	0.83
At least 1 target lesion < 2.25 mm	97 (20.4)	54 (24.1)	43 (17.1)	0.06
Postdilation performed	356 (74.9)	186 (74.1)	170 (75.9)	0.65
Multivessel treatment	93 (19.6)	44 (19.6)	49 (19.5)	0.97

Data are *n*/*N* (%) or mean (SD) in continuous variables

*ZES* zotarolimus-eluting stent, *SES* sirolimus-eluting stent

### Clinical outcomes

**Fig. 1:**
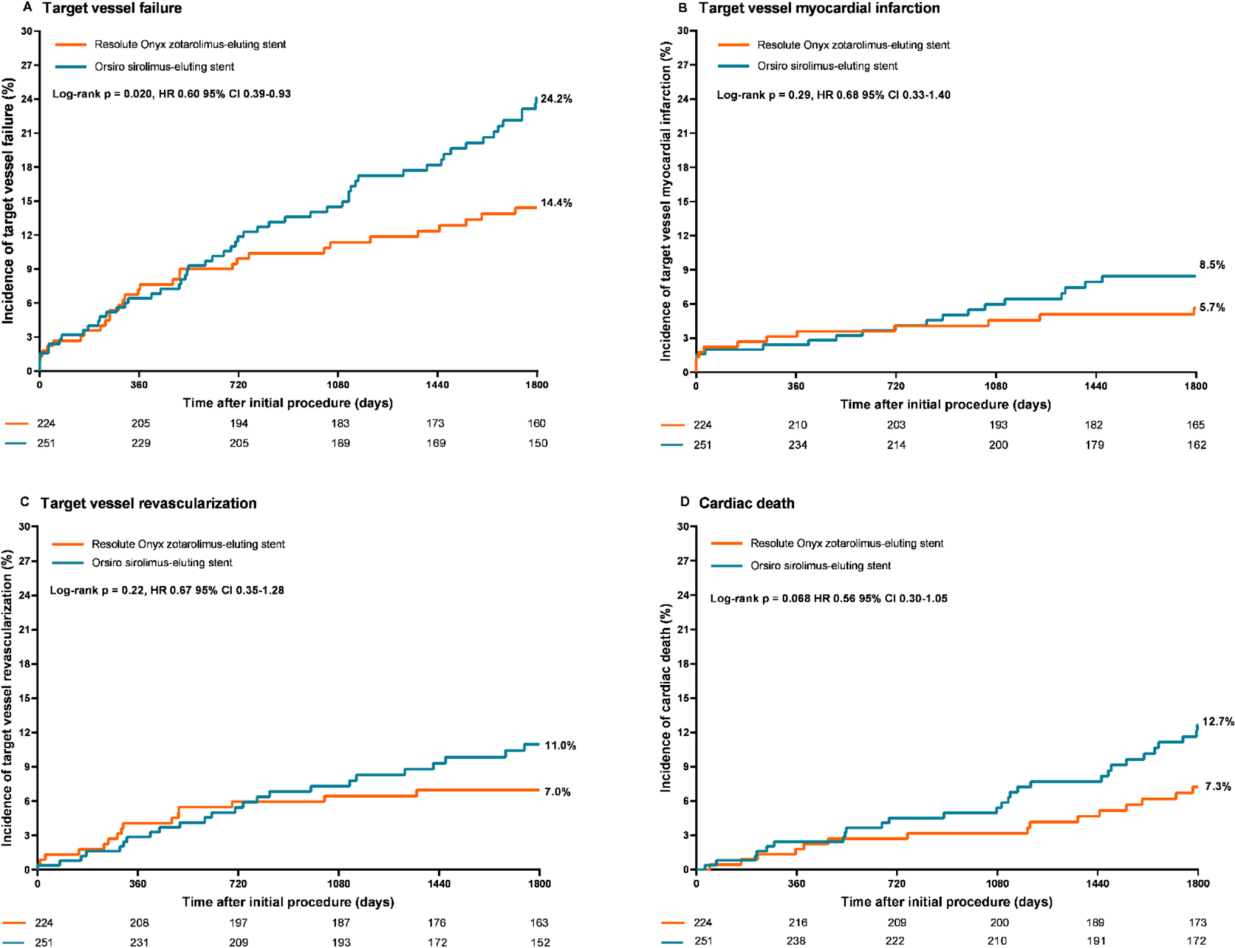
Kaplan–Meier time to event curves of the incidence of the composite main clinical endpoint target vessel failure and its components. *HR* hazard ratio, *CI* confidence interval

After 5-year follow-up, outcome data were available for 461/475 (97.1%) of the elderly patients. Two patients were lost to follow-up and twelve withdrew their consent (0.4% and 2.5%, respectively) (Supplementary Fig. 1). The 5-year rate of the main endpoint TVF was significantly lower in elderly patients treated with Onyx ZES as compared to Orsiro SES (14.4% vs. 24.2%; HR: 0.60, 95% CI 0.39–0.93, *p* = *0.020*). Cardiac death showed a statistically non-significant trend towards a lower rate in patients treated with Onyx ZES (7.3% vs. 12.7%, HR: 0.56, 95% CI 0.30–1.05, p = 0.068). The other individual components of the main endpoint, that is TVMI and TVR, also showed numerically lower rates in patients treated with Onyx ZES (5.7% vs. 8.5%, p = 0.29; and 7.0% vs. 11.0%, p = 0.22, respectively). Figure 1 displays the Kaplan–Meier time-to-event curves of the primary endpoint and its components.

The secondary endpoints MACE and POCE showed a significant difference in favour of Onyx ZES (28.6% vs. 38.7%, *p* = *0.020;* and 30.4% vs. 42.3%, *p* = *0.007*, respectively). In addition, the incidence of death from any cause was significantly lower in patients treated with Onyx ZES (20.5% vs. 28.9%, *p* = *0.030*). The incidence of definite stent thrombosis over the 5-year period was low, and there was no significant difference between the two stent groups (1.0% vs. 1.7%, p = 0.47). Further clinical outcomes are presented in Table 2. Adjustment for sex and diabetes in the multivariate analysis did not change the outcomes (Supplementary Table 3).

**Table 2:** Clinical outcomes at 5-year follow-up and between 1 and 5 years

	Onyx ZES *n* = 224	Orsiro SES *n* = 251	Hazard ratio (95% CI)	P _logrank_
At 5-year follow-up				
Target vessel failure	31 (14.4)	55 (24.2)	0.60 (0.39–0.93)	*0.020*
Target lesion failure	30 (14.1)	52 (22.8)	0.61 (0.39–0.95)	*0.028*
Major adverse cardiac event	63 (28.6)	95 (38.7)	0.69 (0.50–0.94)	*0.020*
Patient-oriented composite endpoint	67 (30.4)	104 (42.3)	0.66 (0.48–0.89)	*0.007*
Death from any cause	45 (20.5)	71 (28.9)	0.66 (0.46–0.96)	*0.030*
Cardiac death	15 (7.3)	28 (12.7)	0.56 (0.30–1.05)	0.068
Vascular death	3 (1.5)	9 (4.0)	0.36 (0.10–1.32)	0.11
Cardiovascular death	18 (8.6)	37 (16.2)	0.51 (0.29–0.90)	*0.018*
Non-cardiovascular death	27 (13.0)	34 (15.2)	0.83 (0.50–1.37)	0.45
Any myocardial infarction	18 (8.8)	27 (12.0)	0.70 (0.39–1.28)	0.24
Target vessel myocardial infarction	12 (5.7)	19 (8.5)	0.68 (0.33–1.40)	0.29
Any revascularization	23 (11.0)	37 (16.4)	0.65 (0.39–1.10)	0.11
Target vessel revascularization	15 (7.0)	24 (11.0)	0.67 (0.35–1.28)	0.22
Target lesion revascularization revascularization	13 (6.1)	20 (9.1)	0.70 (0.35–1.40)	0.31
Definite-or-probable stent thrombosis	2 (1.0)	4 (1.7)	0.54 (0.10–2.94)	0.47
Definite stent thrombosis	2 (1.0)	4 (1.7)	0.54 (0.10–2.94)	0.47

Between 1- and 5-year follow-up				
Target vessel failure	15 (7.8)	39 (18.9)	0.40 (0.22–0.72)	*0.002*
Target lesion failure	14 (7.4)	38 (18.3)	0.38 (0.20–0.69)	*0.001*
Major adverse cardiac event	43 (21.6)	76 (33.6)	0.57 (0.40–0.83)	*0.003*
Patient-oriented composite endpoint	47 (23.5)	78 (35.5)	0.60 (0.42–0.86)	*0.005*
Death from any cause	37 (17.5)	59 (25.4)	0.65 (0.43–0.98)	*0.037*
Cardiac death	11 (5.7)	22 (10.5)	0.52 (0.25–1.07)	0.068
Vascular death	2 (1.0)	6 (2.8)	0.35 (0.07–1.74)	0.18
Cardiovascular death	13 (6.5)	28 (13.0)	0.48 (0.25–0.93)	*0.026*
Non-cardiovascular death	24 (11.8)	31 (14.2)	0.80 (0.47–1.36)	0.41
Any myocardial infarction	10 (5.3)	19 (9.0)	0.54 (0.25–1.17)	0.11
Target vessel myocardial infarction	5 (2.6)	13 (6.2)	0.40 (0.14–1.13)	0.072
Any revascularization	13 (6.7)	24 (11.6)	0.55 (0.28–1.08)	0.08
Target vessel revascularization	6 (3.0)	17 (8.3)	0.37 (0.15–0.94)	*0.029*
Target lesion revascularization	4 (2.1)	15 (7.1)	0.28 (0.09–0.84)	*0.015*
Definite-or-probable stent thrombosis	1 (0.6)	3 (1.3)	0.35 (0.04–3.39)	0.34
Definite stent thrombosis	1 (0.6)	3 (1.3)	0.35 (0.04–3.39)	0.34

Data are *n*/*N* (%)

*ZES* zotarolimus-eluting stent, *SES* sirolimus-eluting stent

The 475 study patients were treated in 638 lesions. Lesion-based data are presented in Supplementary Table 4. There was no between-group difference in lesion characteristics other than a difference in preprocedural reference vessel diameter with smaller dimensions in the Onyx ZES group (2.74 mm vs. 2.83 mm, p = 0.038).

### Landmark analysis between 1- and 5- year follow-up

**Fig. 2:**
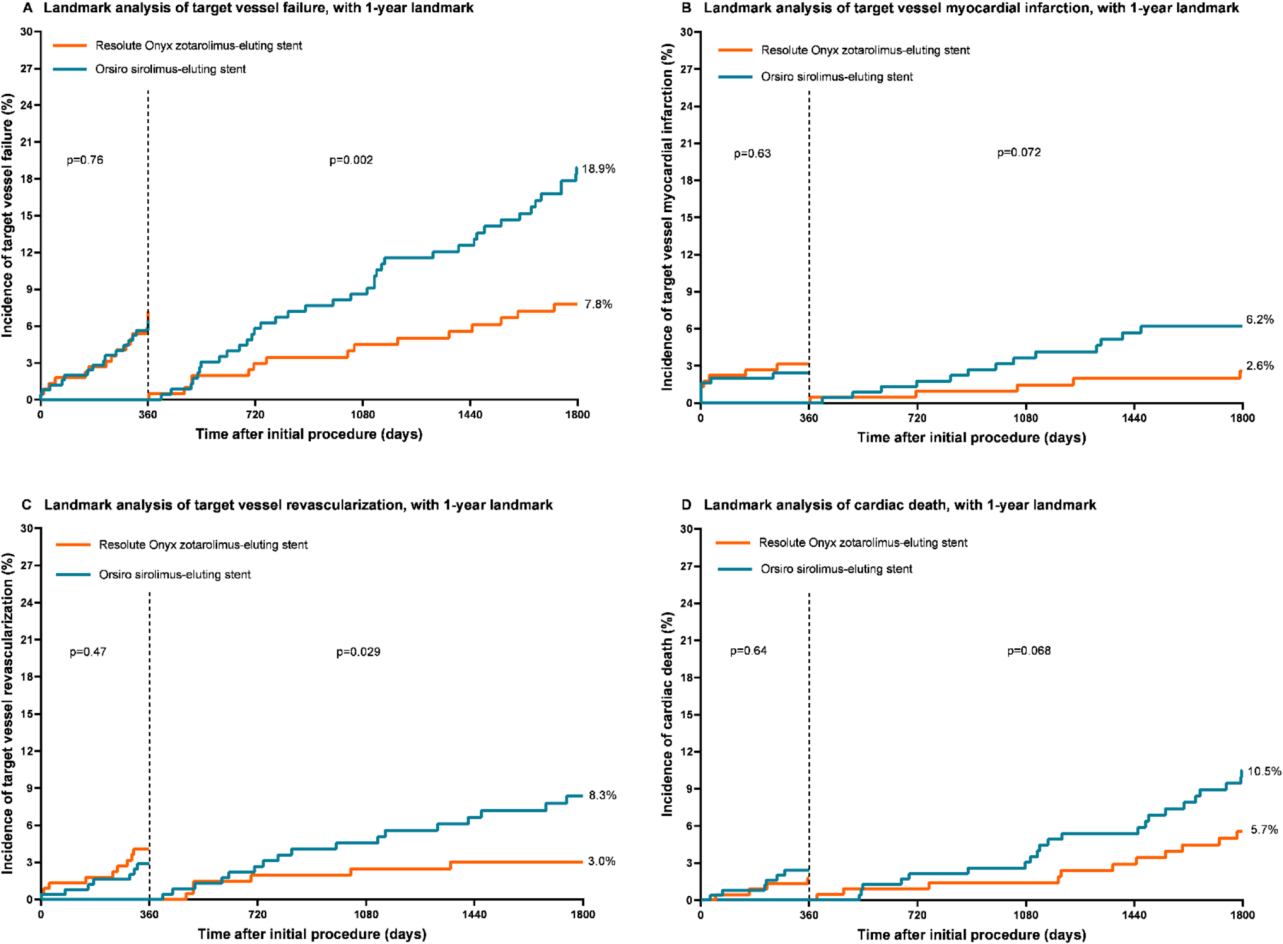
Kaplan–Meier time to event curves of the incidence of target vessel failure and its individual components with a 1-year landmark. P values are P log-rank. *HR* hazard ratio, *CI* confidence interval

The results of a landmark analysis are presented in Table 2. Between 1- and 5-year follow-up, there was a significant lower risk of TVF with the use of Onyx ZES (HR 0.40, 95% CI 0.22–0.72, *p* = *0.002*). Kaplan–Meier time-to-event curves for the main endpoint TVF and its components are presented in Fig. 2. In parallel with the findings at 5-year follow-up, the landmark analysis showed for the secondary endpoints TLF, MACE, and POCE significant between-stent differences in favor of Onyx ZES (*p* = *0.001*, *p* = *0.003*, and *p* = *0.005*, respectively). In addition, between 1- and 5-year follow-up, patients treated with Onyx ZES had lower TVR and target lesion revascularization rates than those treated with Orsiro SES (3.0% vs. 8.3%, p = *0.029;* and 2.1% vs. 7.1%, *p* = *0.015*, respectively).

## Discussion

### Main findings

The present analysis compared long-term clinical outcomes after PCI with two new-generation DES in an elderly patient population, aged 75 years and older. In a pre-specified, secondary analysis of a major randomized all-comer stent trial [13], we evaluated the safety and efficacy of the durable polymer-coated Onyx ZES versus the biodegradable polymer-coated Orsiro SES. At 5-year follow-up, the main composite endpoint TVF showed a significant between-stent difference in favor of the Onyx ZES, reflecting perceivable, yet not statistically significant between-stent dissimilarities in the course of the time-to-event curves of all TVF-components (TVR, TVMI, and cardiac death). A landmark analysis showed in Onyx ZES-treated patients significantly lower rates of TVF and TVR, emerging after the first year of follow-up. In addition, the 5-year incidences of TLF, MACE, POCE, and all-cause mortality were significantly lower in patients treated with Onyx ZES.

### Previous studies in elderly patients

There has been substantial improvement in clinical outcome after PCI in parallel with the refinement of coronary stents: from the first bare metal devices and early-generation DES to the more biocompatible new-generation DES favorable short- and long-term results have been shown [18, 21–27]. The European Society of Cardiology (ESC) guidelines on myocardial revascularization dedicate a small section to PCI in the elderly patient population, which suggests to preferably use new-generation DES instead of bare metal stents [28]. The ESC recommendation for elderly PCI patients is mainly based on the randomized SENIOR trial that assessed 1-year clinical outcomes after PCI with DES in patients aged 75 years or older [29].

To our knowledge, the present study is the first to compare 5-year clinical outcomes with two types of new-generation DES in elderly PCI patients. As a result, most of the study data cannot be directly compared with results of previous studies. A few previous studies with a short follow-up of 1 year support that PCI with new-generation DES is safe and efficacious in elderly patients. A previous patient-level pooled analysis from the TWENTE trials compared the safety and efficacy of PCI with DES in a group of 671 octogenarians and 8,533 patients aged < 80 years [6]. At 1-year follow-up, the rates of the main endpoint TVF were significant higher in octogenarians (7.3% vs. 5.3%, *p* = *0.04*), driven by cardiac death. Since there was no significant difference between the two age categories in myocardial infarction and repeat coronary revascularization, treatment with new-generation DES in octogenarians appears to be safe and efficacious. Yet, in that study, patients were treated with 7 different types of DES, follow-up duration was shorter (1 year vs. 5 years), and no between-stent analysis was performed. The multicenter e-Ultimaster registry analyzed in a substudy a total of 457 elderly patients (≥ 80 years) with STEMI and compared their 1-year clinical outcomes with that of younger patients [5]. Octogenarians had a higher incidence of the primary composite endpoint TLF (7.1% vs. 3.8%, *p* < *0.001*), driven by cardiac death (6.1% vs. 1.6%, *p* < *0.001*). The rates of TVMI 1.2% vs. 0.7%, p = 0.26) and TVR (1.6% vs. 2.0%, p = 0.51) did not differ between-groups [5].

A registry from South Korea investigated the clinical outcomes of 650 elderly patients (≥ 75 years) who underwent PCI with the bio-absorbable polymer-coated everolimus-eluting Synergy stent for various clinical syndromes with the exception of STEMI [30]. After 1 year of follow-up, implantation of the Synergy stent resulted in low device-related clinical outcomes. With regard to the secondary endpoints TVMI and TVR, very low event rates were observed (0.3% and 0.9%, respectively) [23]. These event rates at 1-year are lower than the corresponding TVMI and TVR rates obtained from the landmark analysis in our study that ranged for the two stent groups from 2.4–3.1% and from 2.9–4.1%, respectively. Difference in study design and patient population may partially account for this difference. In addition, low clinical event rates have been consistently observed in other East Asian patient populations and have been attributed to the potential presence of ethnic or genetic protective factors [31]. Furthermore, the TVR rates (6.1% and 9.1%) in elderly patients of the present analysis are consistent with the findings of a pooled analysis of patient-level data from 32,524 PCI trial participants (of various ages) [32].

### Stent design and PCI in elderly patients

As the present study found lower rates of the primary endpoint TVF and several secondary endpoints in patients treated with the Onyx ZES, the question arises as to whether there may be a rationale for that. As a matter of fact, elderly patients more frequently have extensive and complex coronary disease with severely calcified target lesions [7]. Age-related cardiovascular changes, such as endothelial dysfunction, chronic vascular inflammation, and vascular stiffness, promote the progression of coronary artery disease [3, 33]. In the current study, 79% of the elderly patients had at least one complex target lesion and almost 25% had a severely calcified target lesion. The various available DES types differ in several device characteristics such as stent design, polymer coating, and the type of drug and the pharmacodynamics of drug release. Certain of these DES-specific features may benefit the elderly. In the present study, the Onyx ZES has slightly thicker struts with a different strut shape and material than its comparator, which might contribute to a stronger radial force of the Onyx ZES [34]. Due to the higher prevalence of calcified lesions in the elderly, a strong radial stent force and high device flexibility may be beneficial. The latter could be of additional value when performing PCI in calcified lesions, as a more flexible stent is likely to reduce the risk (or extent) of stent mal-apposition. A standardized bench test evaluation of coronary stents, assessing the biomechanical characteristics of DES, found the Onyx ZES to be more flexible than the Orsiro SES [35]. In that study, flexibility was particularly high for the Onyx ZES. This stent feature may also be useful when treating small-vessel lesions. As shown in Supplementary Table 4, our patients treated with Onyx ZES had target lesions with a significantly smaller mean reference diameter than the patients of the Orsiro SES group (2.74 mm vs. 2.83 mm).

In addition, the smallest available stent diameter measured 2.0 mm for the Onyx ZES and 2.25 mm for the Orsiro SES. Typically, PCI with smaller stents have been associated with an increased risk of TVR [36]. Nevertheless, in the present study, in which patients of the Onyx ZES group had both a smaller reference vessel size and the smallest stent size available, the incidence of TVR was found to be lower in the Onyx ZES group as compared to the Orsiro SES group; this is shown in a Landmark analysis after 1-year follow-up (Fig. 2). We can only hypothesize that treatment of lesions in small coronary vessels with smaller stents may lower the risk of lesion recurrence and TVR. Despite the between-DES differences in smallest available stent size and strut thickness, there was no significant between-stent difference in the 5-year rate of stent thrombosis which was very low in both Onyx ZES and Orsiro SES (1.0% vs. 1.7%).

Another unique feature of the Onyx ZES is its platinum-iridium core that results in increased radiopacity of the device [37]. This feature can facilitate PCI procedures in the setting of limited x-ray visibility (e.g., in calcified lesions), which might increase the likelihood of detecting stent under-expansion or mal-apposition. This could have increased the use of high-pressure post-dilatations with slightly oversized, non-compliant balloons. In the present study, stent postdilatation was performed in 75% of the patients in both DES group. This lack of difference in postdilatation between both DES, might have resulted from the wide use of high-pressure stent postdilatation. Theoretically, this may have prevented possible between-group differences in the rate of postdilatation that (otherwise) could have been triggered by differences in the visualization of suboptimal initial results after the stent implantation.

### Limitations

This prespecified secondary analysis of a randomized trial in PCI all-comers assessed the long-term clinical outcome in the important subgroup of elderly patients. Yet, the present study is formally not powered for assessing subgroups, secondary endpoints, or landmark analyses. So, this secondary analysis may lack statistical robustness, and the study findings should be considered as hypothesis-generating only. The sample size was reasonable but not large. Nevertheless, the present analysis of 5-year outcome data is in the range of most previous studies in elderly PCI patients, yet those studies followed the patients for no more than 12 months [5, 6, 30]. The follow-up rate was high (97.1% at 5 years), but registration-based randomized trials may have even higher follow-up rates, yet at the price of other methodological limitations. In routine clinical practice, stent postdilatation was mostly done with non-compliant balloons. Nevertheless, in this study, information on the type of balloon catheter used for stent postdilatation was available only for patients treated at the main enrolling study site. Lastly, the study population contains predominantly Western Europeans [38], which limits the generalizability of the results to broader and more ethnically diverse patient populations.

## Conclusions

In elderly all-comer patients, those treated with Onyx ZES showed a lower 5-year incidence of the main safety and efficacy endpoint, as well as several secondary endpoints, than patients treated with Orsiro SES. Further research on this issue is warranted.

## Supplementary Information

Below is the link to the electronic supplementary material.

Supplementary file1 (DOCX 64 KB)

## Data Availability

Data may be made available upon request in adherence to transparency conventions in medical research and through reasonable requests to the corresponding author, following approval by the steering committee.

## Abbreviations

DES: Drug-eluting stent
MACE: Major adverse cardiac events
PCI: Percutaneous coronary intervention
POCE: Patient-oriented composite endpoint
SES: Sirolimus-eluting stent
STEMI: ST-segment elevation myocardial infarction
TLF: Target lesion failure
TVF: Target vessel failure
ZES: Zotarolimus-eluting stent
