# Supplementary material for: Elderly patients treated with Onyx versus Orsiro drug-eluting coronary stents in a randomized clinical trial with long-term follow-up
Source: Clin Res Cardiol. 2025 Mar 4;114(12):1681–91. doi: 10.1007/s00392-025-02622-7 (PMC12708834; doi:10.1007/s00392-025-02622-7)
Supplement: Supplementary file 1 — Supplementary file1 (DOCX 64 KB) [file 392_2025_2622_MOESM1_ESM.docx]

**Supplemental Material**

**Elderly Patients Treated with Onyx versus Orsiro Drug-eluting Coronary Stents in a Randomized Clinical Trial with Long-term Follow-up**

Daphne van Vliet, MD^1^; Eline H Ploumen, MD PhD^1,2^; Tineke H Pinxterhuis, MD^1,2^;Carine JM Doggen, PhD^2^; Adel Aminian, MD^3^; Carl E Schotborgh, MD^4^; Peter W Danse, MD PhD^5^; Ariel Roguin, MD PhD^6^;

Rutger L Anthonio MD PhD^7^; Edouard Benit, MD^8^; Marlies M Kok, MD PhD^1^;

Gerard CM Linssen, MD PhD^9^, Clemens von Birgelen, MD PhD^1,2^

Supplementary Table 1

**Table 1. Antiplatelet therapy and use of anticoagulants**

Supplementary Table 2

**Table 2. Extended procedural characteristics**

Supplementary Table 3

**Table 3. Clinical outcomes at 5-year follow-up, after multivariate analysis**

Supplementary Table 4

**Table 4. Lesion-based data, p value corrected for intra-patient correlation**

Supplementary Figure 1

**Figure 1. Study flowchart**

**Supplementary Table 1. Antiplatelet therapy and use of anticoagulants**

|  | | | |
| --- | --- | --- | --- |
|  | **Onyx ZES**  **n=224^*^** | **Orsiro SES**  **n=251^±^** | **P_Logrank_** |
| At discharge | | | |
| Aspirin | 215 (96.0) | 234 (93.2) | 0.19 |
| Dual antiplatelet therapy  Clopidogrel  Ticagrelor / Prasurgrel | 215 (96.0)  130 (58.0)  85 (37.9) | 234 (93.2)  139 (55.4)  95 (37.8) | 0.19  0.56  0.98 |
| Vitamin K antagonist (OAC) | 34 (15.2) | 32 (12.7) | 0.47 |
| Direct oral anticoagulant (DOAC) | 13 (5.8) | 14 (5.6) | 0.92 |
| P2Y12 inhibitor + (D)OAC | 47 (21.0) | 46 (18.3) | 0.47 |
| Triple therapy^↑^ | 38 (17.0) | 33 (13.1) | 0.24 |
| At 5-year follow-up | | | |
| Aspirin | 138 (61.6/69.0) | 139 (55.4/68.1) | 0.85 |
| P2Y12 inhibitor | 32 (14.3/16.0) | 38 (15.1/18.6) | 0.49 |
| Dual antiplatelet therapy | 16 (7.1/8.0) | 21 (8.4/10.3) | 0.42 |
| Vitamin K antagonist (OAC) | 16 (7.1/8.0) | 14 (5.6/6.9) | 0.66 |
| Direct oral anticoagulant (DOAC) | 27 (12.1/13.5) | 36 (14.3/17.6) | 0.25 |

Numbers are n/N (%). ^↑^Triple therapy = Aspirin, P2Y12 inhibitor and (N)OAC. P2Y12 inhibitor = clopidogrel, ticagrelor or prasugrel. ^*,±^In 24 Onyx and 47 Orsiro patients, data on the use of anticoagulant and antiplatelet therapy were missing at 5-year follow-up. ZES: zotarolimus-eluting stent, SES: sirolimus-eluting stent.

**Supplementary Table 2. Extended procedural characteristics**

|  | | | | |
| --- | --- | --- | --- | --- |
|  | **All patients**  **n=475** | **Onyx ZES**  **n=224** | **Orsiro SES**  **n=251** | **P _value_** |
| Predilatation | 381 (80.2) | 175 (78.1) | 206 (82.1) | 0.28 |
| Postdilatation* | 356 (74.9) | 186 (74.1) | 170 (75.9) | 0.65 |
| Intravascular ultrasound | 7 (1.5) | 3 (1.3) | 4 (1.6) | 0.82 |
| Optical coherence tomography | 3 (0.6) | 2 (0.9) | 1 (0.4) | 0.50 |
| Fractional Flow Reserve Use | 53 (11.2) | 22 (9.8) | 31 (12.4) | 0.38 |
| Rotablation | 13 (2.7) | 4 (1.8) | 9 (3.6) | 0.23 |
| Cutting balloon | 12 (2.5) | 5 (2.2) | 7 (2.8) | 0.70 |
| Guiding catheter extension | 64 (13.5) | 27 (12.1) | 37 (14.7) | 0.39 |

Data are n/N (%). ZES: zotarolimus-eluting stent, SES: sirolimus-eluting stent. *Of the elderly patients treated in the main enrolling center, 94.3% of the postdilatation was done with non-compliant balloons.

**Supplementary Table 3. Clinical outcomes at 5-year follow-up**

**After multivariate analysis for sex and diabetes**

|  | | | | |
| --- | --- | --- | --- | --- |
|  | **Onyx ZES**  **n= 224** | **Orsiro SES**  **n= 251** | **Adjusted HR (95% CI)** | **P _value_** |
| Target vessel failure | 31 (14.4) | 55 (24.2) | 0.59 (0.38-0.91) | *0.018* |
| Target lesion failure | 30 (14.1) | 52 (22.8) | 0.60 (0.38-0.94) | *0.024* |
| Major adverse cardiac event | 63 (28.6) | 95 (38.7) | 0.68 (0.49-0.93) | *0.016* |
| Patient-oriented composite endpoint | 67 (30.4) | 104 (42.3) | 0.65 (0.48-0.86) | *0.006* |
| Death from any cause | 45 (20.5) | 71 (28.9) | 0.66 (0.45-0.95) | *0.027* |
| Cardiac death | 15 (7.3) | 28 (12.7) | 0.54 (0.29-1.02) | 0.057 |
| Vascular death | 3 (1.5) | 9 (4.0) | 0.36 (0.10-1.32) | 0.12 |
| Cardiovascular death | 18 (8.6) | 37 (16.2) | 0.50 (0.28-0.88) | *0.016* |
| Non-cardiovascular death | 27 (13.0) | 34 (15.2) | 0.83 (0.50-1.38) | 0.47 |
| Any myocardial infarction | 18 (8.8) | 27 (12.0) | 0.69 (0.38-1.27) | 0.24 |
| Target vessel myocardial  infarction | 12 (5.7) | 19 (8.5) | 0.66 (0.32-1.35) | 0.25 |
| Any revascularization | 23 (11.0) | 37 (16.4) | 0.65 (0.39-1.09) | 0.10 |
| Target vessel  revascularization | 15 (7.0) | 24 (11.0) | 0.65 (0.34-1.24) | 0.19 |
| Target lesion  revascularization | 13 (6.1) | 20 (9.1) | 0.69 (0.34-1.38) | 0.29 |
| Definite-or-probable stent thrombosis | 2 (1.0) | 4 (1.7) | * |  |
| Definite stent thrombosis | 2 (1.0) | 4 (1.7) | * |  |

***** Event count too low to perform multi-variate analysis. ZES: zotarolimus-eluting stent, SES: sirolimus-eluting stent.

**Supplementary Table 4. Lesion-based data, p value corrected for intra-patient correlation**

|  | | | | |
| --- | --- | --- | --- | --- |
|  | **All lesions**  **n= 638** | **Onyx ZES**  **n= 307** | **Orsiro SES**  **n= 331** | **P value*** |
| Complex lesion | 466 (73.2) | 226 (73.6) | 240 (72.7) | 0.64 |
| Bifurcation | 228 (34.9) | 105 (34.2) | 123 (37.2) | 0.24 |
| Chronic total occlusion | 17 (2.7) | 7 (2.3) | 10 (3.0) | 0.72 |
| Bypass graft | 17 (2.7) | 9 (2.9) | 8 (2.4) | 0.95 |
| Severely calcified lesion | 141 (22.1) | 78 (25.4) | 63 (19.0) | 0.34 |
| Assigned stents only | 628 (98.4) | 305 (99.3) | 323 (97.6) | 0.057 |
| Total number of stents | 1.30 ± 0.61 | 1.33 ± 0.62 | 1.28 ± 0.60 | 0.45 |
| Postdilation | 429 (67.5) | 213 (69.4) | 216 (65.3) | 0.69 |
| Lesion success | 630 (99.4) | 306 (99.7) | 324 (99.1) | 0.37 |
| Device success | 621 (97.9) | 304 (99.0) | 317 (96.9) | 0.062 |
| Preprocedural data | | | | |
| Lesion length, mm | 15.4 (11.1-23.7) | 15.3 (11.0-23.1) | 15.5 (11.1-24.1) | 0.55 |
| Minimum lumen diameter, mm | 0.77 (0.54-1.07) | 0.76 (0.54-1.07) | 0.78 (0.54-1.06) | 0.80 |
| Reference vessel diameter, mm | 2.79 (0.57) | 2.74 (0.6) | 2.83 (0.56) | *0.038* |
| Lumen diameter stenosis, % | 70.0 (61.1-80.6) | 70.0 (60.3-79.9) | 70.4 (61.5-81.0) | 0.44 |
| Postprocedural data | | | | |
| Maximum pressure postdilatation, mmHg | 19.42 (4.9) | 19.72 (5.2) | 19.13 (4.6) | 0.24 |
| Minimum lumen diameter, mm | 2.39 (0.5) | 2.38 (0.5) | 2.40 (0.5) | 0.44 |
| Reference vessel diameter, mm | 2.76 (0.5) | 2.74 (0.6) | 2.78 (0.5) | 0.18 |
| Lumen diameter stenosis, % | 12.5 (8.1-18.6) | 12.6 (7.9-18.1) | 12.4 (8.1-18.9) | 0.27 |
| Acute lumen gain in segment, mm | 1.61 (0.6) | 1.59 (0.6) | 1.63 (0.6) | 0.37 |

Lesion success defined as restlesion <50%. Device success defined as restlesion <50% and assigned stents only. *P values were corrected for intra-patient correlation as patients could have more than one target lesion. ZES: zotarolimus-eluting stent, SES: sirolimus-eluting stent.

**Supplementary Figure 1. Study flowchart**


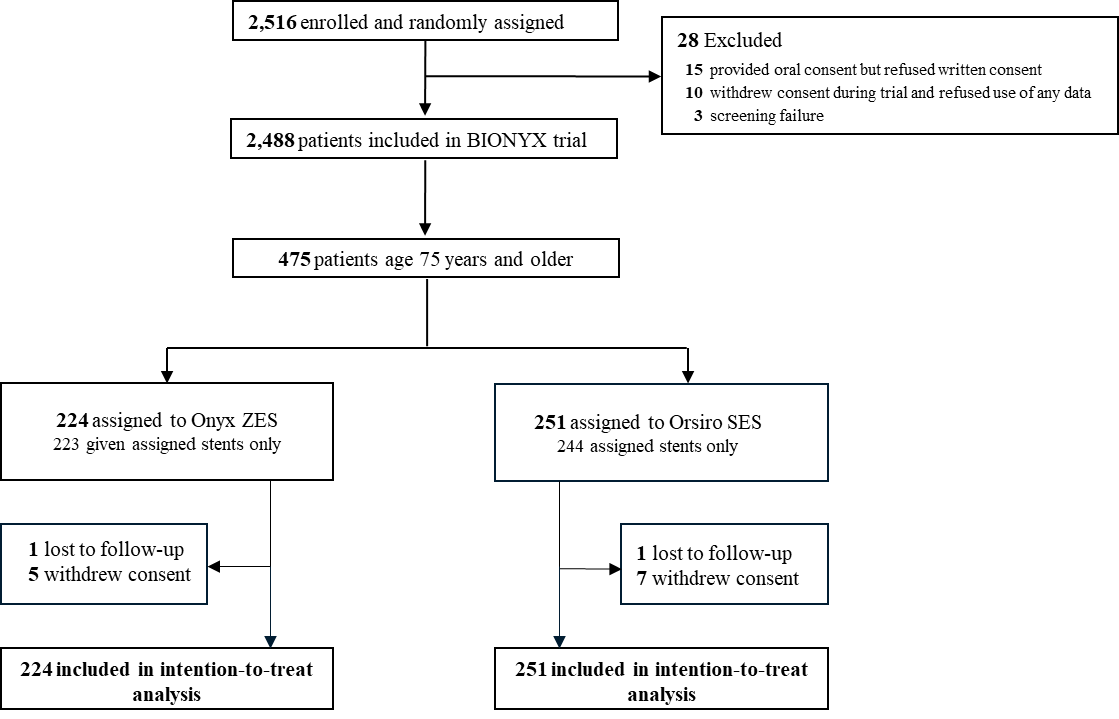


*Abbreviations:* ZES = zotarolimus-eluting stent, SES = sirolimus-eluting stent
